# A Case of Spontaneous Evolution of the Fœtus

**Published:** 1848-04

**Authors:** N. W. Cole

**Affiliations:** Burlington, N. J.


*A case of Spontaneous Evolution of the Fœtus.* By N. W. Cole, M. D., of Burlington, N. J.—On the evening of 10th of October, I received a message from my friend Dr. Haines, to visit with him a female in labor of her eleventh child, to whom he had been called in the afternoon, and to bring with me my instruments. The Doctor informed me that upon his arrival he found one child born, and that another was presenting, with the shoulder and its right arm protruding. He stated also that he had made several unsuccessful attempts to turn. Upon examination I found everything as above stated. The labor pains had entirely ceased, from the time of the birth of the first child. Its waters evacuated, and the uterus was closely and rigidly contracted around its body, so much so that after repeated efforts, I was obliged to abandon all idea of turning without using more force than would be justifiable.

While preparing the necessary instruments for taking the child away, (it was dead beyond all doubt—there being no pulsation from the time of my arrival) the mother's pains returned; and Dr. Haines, who was sitting with her, observed the child to be lower down. I immediately made an examination, and found that some change was taking place. Its body seemed to descend, and I distinctly felt its shoulder ascend. In less than five minutes from the return of the pains, it had made a complete somerset. The feet and breech came down, and the head followed without any difficulty. It was healthy, and of the average size.

Note. We regret that Dr. Cole has not had time to give us a statement of his views upon the rationale of the process which he has described. The case is certainly an interesting one, and we take the liberty of mentioning one or two additional facts which we have obtained from conversation with Drs. Cole and Haines. Upon examination, the head of the child was distinctly felt lying on the left side of the pelvic cavity. The jaw and the ear were readily distinguished, and the hand was so far protruded that there could be no mistake as to the character of the presentation. The breech could not have presented at the os-uteri, and the hand in the vagina, by reason of this extremity lying upon the side of the body, as was the fact in a case reported by Dr. Gooch, or the head and face could not have been so distinctly felt, or the hand found protruding outside the vagina.

The case first reported by Dr. Denman, we believe in 1772, was, as we apprehend, similar to that reported by Dr. Cole and the term "spontaneous evolution" was adopted by that distinguished author. Dr. Douglass, however, in 1811, proposed the term "spontaneous expulsion," as more expressive. Supposing that Dr. Denman's explanation was incorrect, and that the thorax became partially doubled upon itself, and the fœtus expelled while the head remained in its original position, as it were a fixed point; the side of the thorax and abdomen passing rapidly over the perinæum, leaving the head and other arm to complete the last stage of labor. But Dr. Cole informs us, that as the pains increased, "the body seemed to descend, and I distinctly felt its shoulder ascend." This ascent of the shoulder continued until the arm was carried completely up into the uterus, and the breech and feet spontaneously took its place. Here then was a clear and distinct evolution;—to use the graphic term of Dr. C., a "complete somerset;" and after an obstetric practice of half a century, this case has presented to him the first exemplification of Dr. Denman's theory. We gladly give the article a place in our journal, and hope to hear from its author again. Ed.—*New Jersey Med. Reporter.*

